# How Can We Improve Patient-Clinician Communication for Men Diagnosed with Prostate Cancer?

**DOI:** 10.1016/j.euros.2024.01.011

**Published:** 2024-02-13

**Authors:** Katharina Beyer, Ailbhe Lawlor, Sebastiaan Remmers, Carla Bezuidenhout, Juan Gómez Rivas, Lionne D.F. Venderbos, Emma J. Smith, Giorgio Gandaglia, Steven MacLennan, Sara J. MacLennan, Anders Bjartell, Alberto Briganti, Philip Cornford, Susan Evans-Axelsson, Maria J. Ribal, James N'Dow, Erik Briers, Monique J. Roobol, Mieke Van Hemelrijck, for the PIONEER Consortium

**Affiliations:** a Department of Urology, Erasmus MC Cancer Institute, Erasmus University Medical Center Rotterdam, The Netherlands; b Translational Oncology and Urology Research, King’s College London, London, UK; c European Association of Urology Guidelines Office, Arnhem, The Netherlands; d Department of Urology, Clínico San Carlos University Hospital, Madrid, Spain; e Department of Urology, Division of Experimental Oncology, Urological Research Institute, Vita-Salute San Raffaele University, IRCCS San Raffaele Scientific Institute, Milan, Italy; f Academic Urology Unit, University of Aberdeen, Aberdeen, Scotland; g Royal Liverpool and Broadgreen Hospitals NHS Trust, Liverpool, UK; h Department of Translational Medicine, Lund University, Lund, Sweden; i Medical Affairs Oncology, Bayer AB, Stockholm, Sweden; j Patient advocate, Hasselt, Belgium

## Abstract

**Background and objective:**

The ability of health care professionals to communicate with patients compassionately and effectively is crucial for shared decision-making, but little research has investigated patient-clinician communication. As part of PIONEER—an international Big Data Consortium led by the European Association of Urology to answer key questions for men with prostate cancer (PCa), funded through the IMI2 Joint Undertaking under grant agreement 777492— we investigated communication between men diagnosed with PCa and the health care professional(s) treating them across Europe.

**Methods:**

We used the European Organisation for Research and Treatment of Cancer Quality-of-Life Questionnaire-Communication 26, which was shared via the PIONEER and patient organisations on March 11, 2022. We sought men who spoke French, Italian, Spanish, German, Dutch, or English who were diagnosed with PCa and were undergoing or had already received treatment for their PCa.

**Results and limitations:**

A total of 372 men reported that they communicated with their clinician during either the diagnostic or the treatment period. Overall, the majority of participants reported positive experiences. However, important opportunities to enhance communication were identified, particularly with regard to correcting misunderstandings, understanding the patient’s preferred approach to information presentation, addressing challenging questions, supporting the patient’s comprehension of information, attending to the patient’s emotional needs, and assessing what information had already been given to patients about their disease and treatment, and how much of it was understood.

**Conclusions and clinical implications:**

These results help us to identify gaps and barriers to shared treatment decision making. This knowledge will help devise measures to improve patient-health care professional communication in the PCa setting.

**Patient summary:**

As part of the PIONEER initiative, we investigated the communication between men diagnosed with prostate cancer and their health care professionals across Europe. A total of 372 men from six different countries participated in the study. Most participants reported positive experiences, but areas where communication could be improved were identified. These included addressing misunderstandings, tailoring the presentation of information to the patient’s preferences, handling difficult questions, supporting emotional needs, and assessing the patient’s understanding of their diagnosis and treatment.

## Take Home Message

As part of the PIONEER initiative, we conducted a study of patient-clinician communication in the prostate cancer setting in six European countries. Survey responses from 372 patients highlighted areas for improvement, such as clarifying misunderstanding, tailoring information presentation, handling challenging questions, and supporting emotional needs. Efforts to address these issues could help in enhancing patient-clinician communication and shared decision-making for men diagnosed with prostate cancer.

## 1 Introduction

Prostate cancer (PCa) is one of the most commonly diagnosed cancers among men. In Europe, 30 million men are confronted with a diagnosis of PCa in their lifetime [1]. Overall survival rates are high: 83% of men live for more than 5 yr after their diagnosis [2], [3]. Recent improvements in PCa treatment have resulted in prediction of declining mortality rates, but also a lack of consensus on the best treatment [2], [3].

Engaging patients in the decision-making process by enabling them to understand the harms, benefits, and possible outcomes of treatment options, empowers them to choose a treatment that is right for them. This is also known as shared decision-making (SDM). SDM consists of communication, collaboration, aspects of evidence-based medicine, and relationship building. It is based on the principle of the autonomy of the patient. It is a complex process and a unique treatment decision-making model can be implemented for each consultation [4].

When patients and their health care professionals have a relationship characterized by trust and mutual respect [5], patients experience the benefits of SDM. This includes outcomes such as higher satisfaction and confidence in treatment decisions [6], [7], greater levels of treatment adherence [6], [8], higher quality of life [6] and greater coping with the uncertainties of their cancer diagnosis [8]. In addition, patients are less likely to have decisional regret and they report feeling informed or empowered more frequently [5]. Moreover, the patient-clinician relationship has been linked to perception of pain intensity, understanding of information, and psychological adjustment [7]. Conversely, when cancer patients reported that they felt their clinicians were disengaged and less supportive, they experienced higher levels of hopelessness, distress, and maladaptive coping [7].

The ability of clinicians to communicate with patients compassionately and effectively is crucial for SDM. However, adoption of SDM in routine practice across cancer care has been slow and clinicians often find it difficult to accomplish [9], [10], [11]. One of the reasons why SDM may not work in practice is the lack of focus on communication skills during patient-physician consultations [12].

To address this issue, our aim was to gain a better understanding of the communication between men diagnosed with PCa and their treating clinician(s) across Europe to help in optimising the treatment decision-making process.

This project is part of the PIONEER Consortium (Prostate Cancer Diagnosis and Treatment Enhancement Through the Power of Big Data in Europe), an international collaboration led by the European Association of Urology (EAU) that aims to use big data technologies to improve guideline development and clinical practice. One of the major objectives of the consortium was to identify and prioritise the major unanswered questions in the field of PCa. Omar et al [13] conducted a prioritisation exercise to identify which questions are important to patients and health care professionals. Among other questions, understanding the communication between physicians and patients was identified and ranked as important. We therefore addressed this question as part of the present study.

## 2 Patients and methods

To understand the communication between patients and their clinicians in a snapshot across the EU, we used the European Organisation for Research and Treatment of Cancer Quality of Life Questionnaire-Communication 26 (EORTC QLQ-COMU26) module, which was developed for evaluation of interactions between patients and health care professionals. This patient-reported outcome measure (PROM) was chosen as our preferred tool because it is the only PROM developed (and currently validated) in a European population [14]. We transferred the PROM into an online version using Survey Monkey (https://www.surveymonkey.com). The survey was approved as a minimal risk study by the ethics board of King’s College London.

### 2.1 Patient screening criteria

Men were invited to participate in the PIONEER patient-clinician communication survey (COMU26) if they had been diagnosed with PCa and were undergoing or had already received treatment for their PCa. No time interval was added, as this was difficult to control owing to the recruitment method used. Participants were required to speak English, German, Dutch, French, Spanish, or Italian fluently to comprehend the questions. We chose these specific languages because of the visibility of these countries in the PIONEER project.

### 2.2 Recruitment and data collection

We recruited via patient organisations involved with PIONEER, such as Europa Uomo, the EAU patient office, and the European Cancer Patient Coalition. Patient organisations were asked to use their network to promote the survey and encourage participation among PCa patients across Europe. They either shared the survey within their network or added the link to their website to enable patients to participate.

The PIONEER patient-clinician communication survey was accessible in six different languages (English, German, French, Italian, Spanish, and Dutch).

### 2.3 PROM instrument

The EORTC QLQ-COMU26 PROM is applicable across various tumour locations and stages of disease and treatment (including diagnosis, treatment, and follow-up, including palliative care), and focuses on assessing the patient’s communication experiences with different groups of professionals, including doctors, nurses, radiotherapy technicians, and others. Patients are asked to specify the particular treatment phase they were addressing (diagnosis, treatment, or follow-up) [14].

Comprising a total of 26 items, the EORTC QLQ-COMU26 primarily examines communication-related behaviours, organised into six scales and four individual items. The six scales encompass: patient-initiated communication behaviours, aspects of the clinician-patient relationship, qualities of professionals in establishing rapport (we renamed this item “Professional’s qualities in creating a relationship”), skills of professionals, management of patient emotions by professionals, and skills of professionals in delivering information. The four individual items are: professionals considering patient preferences for how information is presented; addressing misunderstandings in information as needed; ensuring privacy; and overall satisfaction with communication.

Descriptive statistics were used to analyse the data. We then grouped findings according to the six scales.

## 3 Results

The survey was shared on March 11, 2022 and closed on August 1, 2023. Overall, 387 patients completed the survey, comprising 72 Dutch, 172 English, five French, seven Italian, 52 German, and 73 Spanish participants. The majority of the participants (266, 72%) reported on communication with their doctor, followed by their nurse (94, 25%), radiotherapy technician (63, 16), psychologist (29, 7.5%), and others (26, 6.7%).

The greatest number of participants (125, 42%) reported on communication with their physician during treatment decisions, but 114 (35%) reported on communication at the time of their diagnosis and 58 (20%) on communication during their follow-up period.

Our initial aim was to assess country-specific differences; however, as the number of responses varied among the countries, we only present total counts for the responses for each item, with percentages calculated using the total number of responses for that item as the denominator.

### 3.1 Patient-initiated communication behaviours

**Fig. 1:**
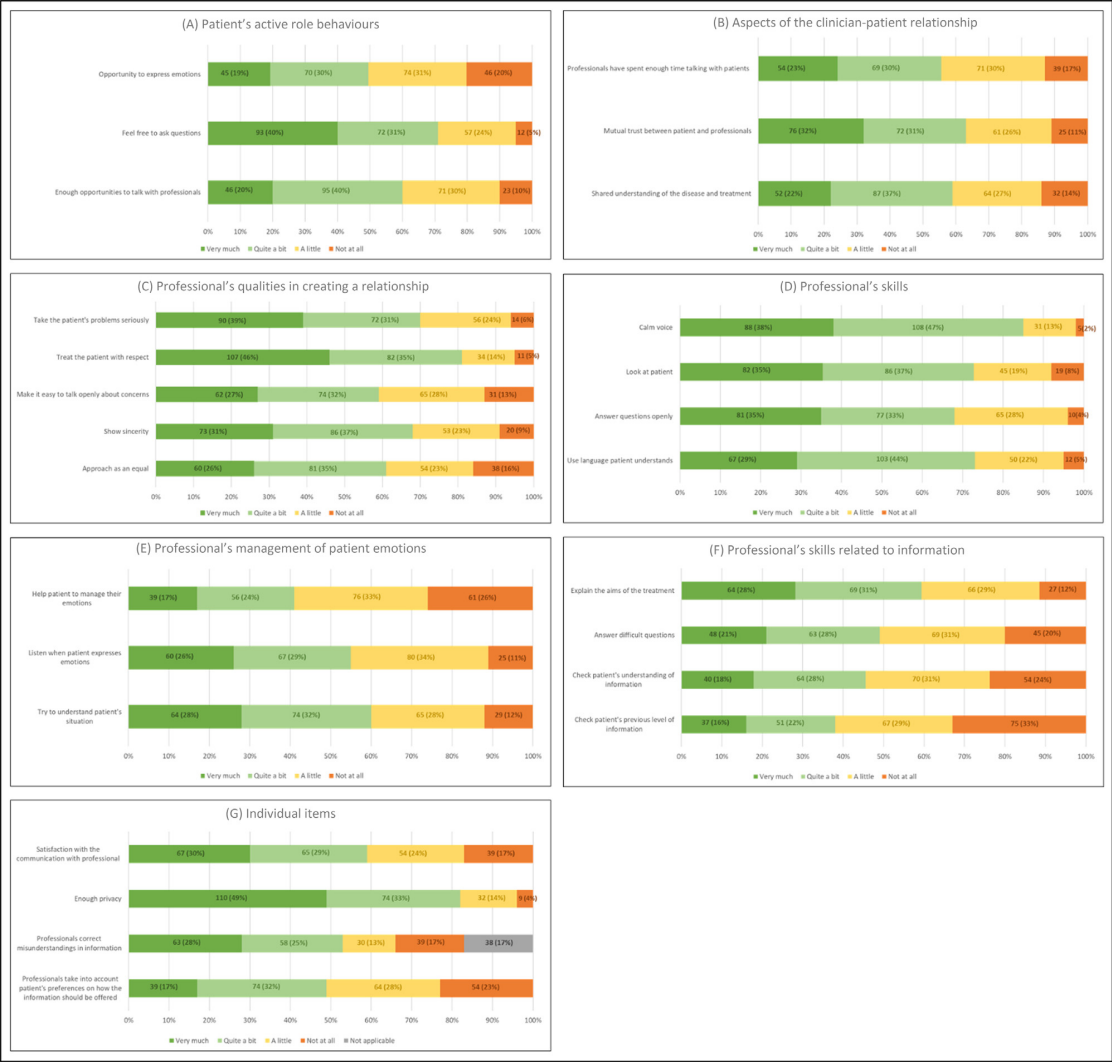
. Results for (A) patient-initiated communication behaviours, (B) aspects of the clinician-patient relationship, (C) professional’s qualities in creating a relationship, (D) skills of professionals, (E) management of patient emotions by professionals, (F) skills of professionals in delivering information, and (G) the four individual items not included in the six scales.

More than half of the respondents (120, 51%) felt they had little to no opportunity to express their emotions. Although 165 (70%) felt free to ask questions, 12 respondents (5%) felt they had no opportunity at all. Moreover, 94 (40%) felt they had little (71, 30%) or no (23, 10%) opportunity to talk with their clinician during diagnosis, treatment, or the follow-up period (Fig. 1A).

### 3.2 Aspects of the clinician-patient relationship

As shown in Figure 1B, 110 respondents (47%) felt that their treating clinician did not have enough time to talk to them, and 68 (37%) felt there was little mutual trust between themselves and their clinician. Moreover, 96 respondents (41%) felt that they had little (64, 27%) or no (32, 14%) shared understanding of the disease and treatment.

### 3.3 Professional’s qualities in creating a relationship

Among the respondents, 189 (81%) reported being treated respectfully and 159 (68%) perceived a sense of genuineness, but only 136 (59%) felt they could openly discuss their apprehensions. Moreover, 70 respondents (30%) indicated that their clinician did not sufficiently acknowledge their concerns and 92 (39%) reported “little” or “not at all” for the item on being approached as an equal (Fig. 1C).

### 3.4 Professional’s skills

Regarding the professional skills of their treating clinician, 196 respondents (85%) reported that the clinician used a calm voice, 168 (72%) that the clinician looked at them, and 170 (73%) that the clinician used language that they could understand. However, 115 respondents (32%) felt that their questions were not answered openly (Fig. 1D).

### 3.5 Management of patient emotions by professionals

Regarding patient emotions, 137 respondents (59%) felt that they were not helped to manage their emotions and 105 (45%) sensed that the clinician was not listening when they tried to express their emotions. Moreover, 94 respondents (40%) felt that the treating clinician did not try to understand their situation (Fig. 1E).

### 3.6 Professional’s skills in delivering information

The treatment objectives remained unclear for 27 respondents (12%), while 64 (28%) felt that they very much understood the aim. More than half of respondents to the question on obtaining answers to complex questions reported that they encountered challenges (114, 51%). In addition, 124 respondents (55%) reported that health care professionals did not enquire about their comprehension of the information conveyed and 142 (62%) reported that they were not asked about their prior exposure to the information (Fig. 1F).

### 3.7 Individual items

Overall, less than half of the respondents reported that they were little (54, 24%) or not at all (39, 17%) satisfied with the communication with their professional. Approximately half of the respondents (121, 53%) reported that if they did not understand something, it was explained by the clinician. Less than half were asked how they prefer to take part in discussion (113, 49%); in other words, their preference regarding how the information should be shared was not considered. Only nine respondents (4%) felt they did not have enough privacy, whereas 184 (82%) were satisfied with the level of privacy during the treatment pathway (Fig. 1G).

## 4 Discussion

**Fig. 2:**
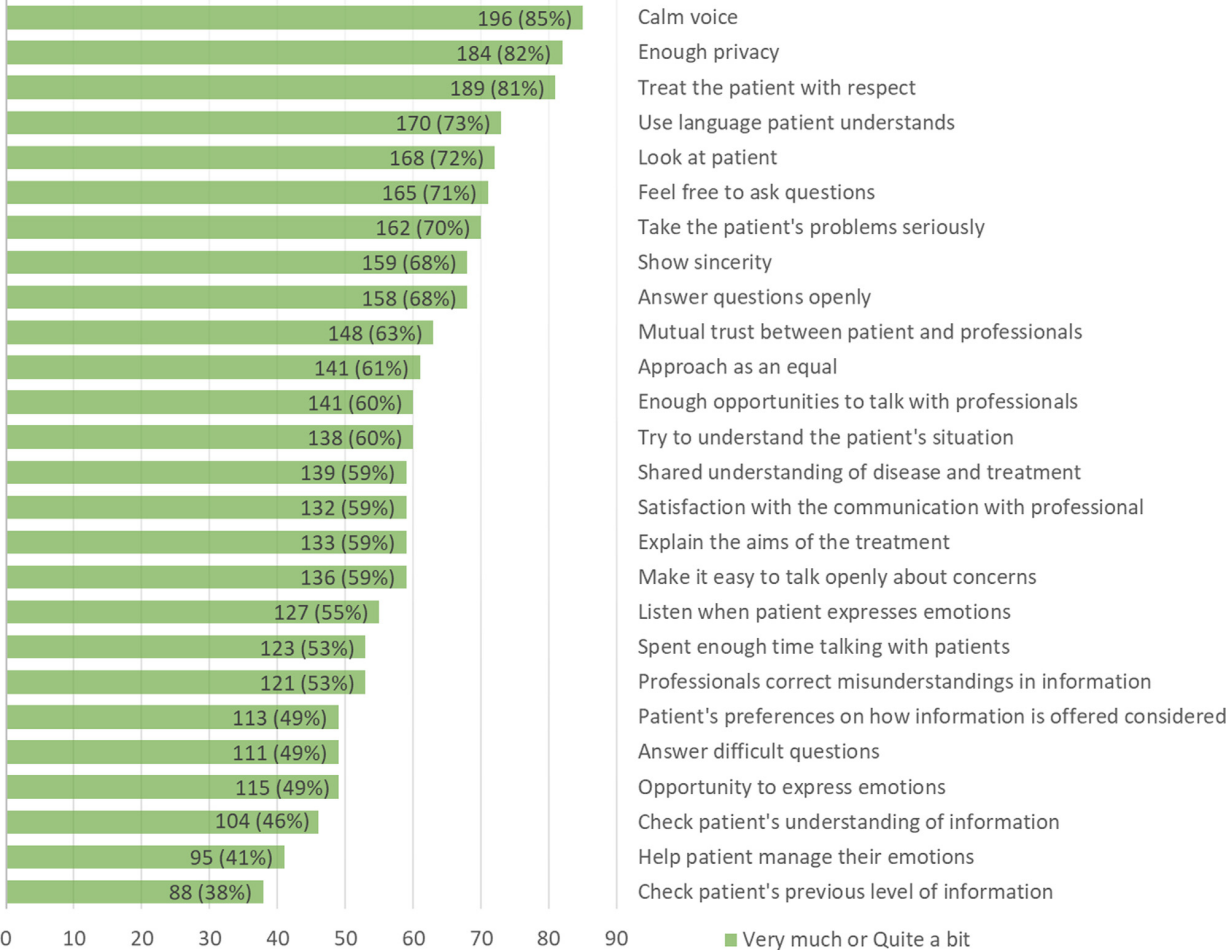
. Complete list of items in the questionnaire ranked by the positive response rate.

To the best of our knowledge, this survey provides the first cross-sectional European perspective on the views of men diagnosed with PCa regarding communication with their health care professionals. Overall, the majority of respondents felt that their clinician communicated with a calm voice (196, 85%), safeguarded their privacy (184, 82%), treated them respectfully (189, 81%), and used language that they could understand (170, 73%). However, important requirements for better communication were identified. In particular, only 53% of respondents stated that their misunderstanding was corrected, only 49% were satisfied that their preferred approach to information presentation was understood and that challenging questions were addressed, only 46% agreed that their comprehension of information was supported, and only 41% agreed that health care professionals attended to their emotional needs. Furthermore, the lowest rating (38%) was for the item on checking the patient’s previous level of knowledge, which highlights a critical need for improvement in establishing what information has already been provided to patients about their disease and treatment and how much of this information they understand (Fig. 2). This is particularly important, as the patient’s preferences must be fully informed to facilitate effective SDM.

Communication is a cornerstone of good patient-clinician relationships and ensures that the patient is included in the decision-making process [15]. Across cancer care, different approaches have been suggested to try to improve communication, with the ultimate goal of adopting SDM as routine in clinical practice. However, our survey results show that several aspects of patient-clinician communication can be improved.

In 2021, Schillinger et al [16] highlighted the need to use a common language during patient-physician interactions. The authors pointed out that it is important to use lay language when explaining a diagnosis, treatment, side effects, and follow-up care, especially for patients with lower health literacy. Research has also shown that actively providing information and asking patients about their illness perception can strengthen patient-clinician communication [15].

Evidence also suggests that decision aids are a solution that can empower patients and help in closing communication gaps during consultations, especially for patients who are less confident in making a decision [17]. Grüne et al [18] assessed the quality of decision aids in 2021 for uro-oncology patients. A conclusion from their systematic review was that the decision aids developed, especially for PCa, are of high quality. However, the effectiveness of the aids can be increased by tailoring them to specific patient needs. These decision aids support knowledge transfer and provide an opportunity for clinicians and patients to address any outstanding questions. A study involving 988 patients by Huber et al [19] demonstrated that overall, the use of decision aids supported guideline adherence, increased health literacy, and enhanced patient autonomy. However, a key component to successful implementation of decision aids is that the decisions need to be followed. A review by Grauman et al [15] revealed that deviation of treatment decisions is often because of the preference of the clinician for more traditional treatment options.

Maskrey [12] highlighted that up to 2019, communication had not been a strong focus either in medical training or in specialised training, and identified this as a contributor to the slow implementation of SDM in clinical practice. Pieterse et al [20] echoed the critique and emphasised that, especially in specialised training, doctors receive a lot of feedback on their technical skills, but communication with patients is almost never supervised. Gulbrandsen [21] added that training in communication skills is currently delivered as a set of instructions, and often overlooks essential lessons about how to navigate conversations in which one person holds more power or influence. This was heavily reflected in our results, where the lowest ranked items are linked to establishing a shared understanding of the information discussed.

Another way to highlight the importance of patient-centred communication has been proposed by the Quality and Outcomes Framework and the pay-for-performance system in the primary care setting in the UK (England). These encourage individualised decision-making by including the ability to record a patient preference as an outcome, such as, “The patient has chosen not to receive the intervention described in the indicator” [12], which could be one way to make patient priorities explicitly visible in clinical records.

### 4.1 Limitations

It is important to acknowledge that the recruitment strategy via patient organisations may have introduced some bias in our population of respondents, as it primarily targeted patients who are active in the groups and may have overlooked the perspectives of patients who are less active in patient organisations. In addition, the response from each country was not proportional, which may potentially skew the analysis in favour of the greatest responding nations. Owing to the recruitment method used, data collection was heavily dependent on the visibility of the recruiting patient organisations in one country. Nonetheless, we believe that this method was the most feasible way to capture a cross-sectional snapshot across Europe, as it provided valuable insights into the perspectives of patients who actively engage in discussions and are likely to be more vocal in sharing their experiences. Future studies should aim to incorporate more diverse recruitment strategies by perhaps targeting a more defined population to ensure a broader representation of patients’ perspectives in one area, while also understanding demographics and cultural differences specific to a country or region.

## 5 Conclusions

Patient-physician communication has been ranked as a research priority by patients and physicians across the EU. Our survey shows that there is little focus on the patient’s emotional needs during consultations and highlights the importance of enabling patients and physicians to have a shared understanding of the topics discussed during consultation. This underscores the need for continued efforts to bridge existing gaps in patient-physician communication. Even though patients seem content about many aspects of patient-physician communication, there are important barriers that currently hinder an SDM environment in clinical practice.

  ***Author contributions***: Katharina Beyer had full access to all the data in the study and takes responsibility for the integrity of the data and the accuracy of the data analysis.

*Study concept and design*: Beyer, Lawlor, S. MacLennan, S.J. MacLennan, Bjartell, Cornford, Evans-Axelsson, Ribal, N’Dow, Briers, Roobol, Van Hemelrijck.

*Acquisition of data*: Bezuidenhout, Gómez Rivas, Venderbos, Smith, Gandaglia, Briers.

*Analysis and interpretation of data*: Beyer, Lawlor, S. MacLennan, S.J. MacLennan, Van Hemelrijck.

*Drafting of the manuscript*: Beyer, Lawlor, Van Hemelrijck, S. MacLennan.

*Critical revision of the manuscript for important intellectual content*: Remmers, Bezuidenhout, Gómez Rivas, Venderbos, Smith, Gandaglia.

*Statistical analysis*: Remmers.

*Obtaining funding*: Evans-Axelsson, Ribal, N’Dow, Bjartell, Cornford.

*Administrative, technical, or material support*: Bezuidenhout.

*Supervision*: S. MacLennan, S.J. MacLennan.

*Other*: None.

  ***Financial disclosures:*** Katharina Beyer certifies that all conflicts of interest, including specific financial interests and relationships and affiliations relevant to the subject matter or materials discussed in the manuscript (eg, employment/affiliation, grants or funding, consultancies, honoraria, stock ownership or options, expert testimony, royalties, or patents filed, received, or pending), are the following: None.

  ***Funding/Support and role of the sponsor:*** PIONEER is funded through the IMI2 Joint Undertaking and is listed under grant agreement 777492. This joint undertaking receives support from the European Union Horizon 2020 research and innovation programme and the European Federation of Pharmaceutical Industries and Associations (EFPIA). The sponsor played a role in the design and conduct of the study, collection, management, analysis, and interpretation of the data, and preparation and review of the manuscript. The views communicated here are those of PIONEER. Neither the Innovative Medicines Initiative nor the European Union, EFPIA, or any associated partner is responsible for any use that may be made of the information contained herein.
